# Social and Economic Characteristics of Street Youth by Gender and Level of Street Involvement in Eldoret, Kenya

**DOI:** 10.1371/journal.pone.0097587

**Published:** 2014-05-14

**Authors:** Rebecca Sorber, Susanna Winston, Julius Koech, David Ayuku, Liangyuan Hu, Joseph Hogan, Paula Braitstein

**Affiliations:** 1 School of Medicine, Indiana University, Indianapolis, Indiana, United States of America; 2 Department of Pediatrics, Warren Alpert School of Medicine, Brown University, Providence, Rhode Island, United States of America; 3 Rhode Island Hospital-Hasbro Youth's Hospital, Providence, Rhode Island, United States of America; 4 AMPATH Consortium, Eldoret, Kenya; 5 Department of Behavioral Sciences, School of Medicine, College of Health Sciences, Moi University, Eldoret, Kenya; 6 Department of Biostatistics, Brown University, Providence, Rhode Island, United States of America; 7 Department of Medicine, School of Medicine, College of Health Sciences, Moi University, Eldoret, Kenya; 8 Dalla Lana School of Public Health, University of Toronto, Toronto, Ontario, Canada; 9 Regenstrief Institute, Inc., Indianapolis, Indiana, United States of America; Harvard School of Public Health, United States of America

## Abstract

**Background:**

Street-connected youth are a neglected and vulnerable population, particularly in resource-constrained settings. The development of interventions and supports for this population requires insight into how they live. This study describes the social and economic characteristics of a convenience sample of street youth (SY) in Eldoret, Kenya.

**Methods:**

Participants were eligible if they were aged 12–21, living in Eldoret, spending days only (part-time), or nights and days on the street (full-time) and able and willing to consent or assent. Data were collected using a standardized interview conducted in English or Kiswahili. Binary dependent variables were having been arrested and/or jailed, and first priority for spending money (food vs. other). Nominal categorical dependent variables included major source of support, and major reason for being street-involved. Multivariable analysis used logistic regression models to examine the association of gender and level of street-involvement with social and economic factors of interest adjusting for age and length of time on the street. Data were analyzed using SAS 9.3.

**Results:**

Of the 200 SY enrolled, 41% were female, mean age of 16.3 years; 71% were on the street full-time, and 29% part-time. Compared with part-time SY, full-time SY were more likely to have been arrested (Adjusted Odds Ratio [AOR]: 2.33, 95% Confidence Interval [95%CI]:1.01–5.35), name food as their first spending priority (AOR: 2.57, 95%CI:1.03–6.45), have left home due to violence (AOR: 5.54, 95%CI: 1.67–18.34), and more likely to report friends on the street as a major source of support (AOR: 3.59, 95% CI: 1.01–12.82). Compared with females, males were more likely to have ever been arrested (AOR: 2.66, 95%CI:1.14–6.18), and to have ever been jailed (AOR: 3.22, 95%CI:1.47–7.02).

**Conclusions:**

These results suggest a high degree of heterogeneity and vulnerability among SY in this setting. There is an urgent need for interventions taking into consideration these characteristics.

## Introduction

The prevalence of children and adolescents (youth) living and working on the streets of the world's cities is a widespread and escalating problem. Due to the transient nature of their lifestyle, estimating the size of street youth populations is difficult [1], but within Kenya alone, the number of street youth has been reported to be as large as 300,000 [2]. However, the label “street youth” implies a homogeneous population and does not have the same meaning and connotation in all locations [1]. Often, street youth are grouped in two major categories: 1) those who live on the streets part-time, often referred to as ‘on the street’, who spend their days on the street but sleep at home with family, are believed to represent approximately 60% of the street youth population in resource-constrained settings; and 2) youth who live on the streets full-time, called ‘of the street’, who spend their days and nights on the street and maintain few or no ties with family, and make up the remaining 40% [3].

Although the full-time versus part-time distinction has historically dominated the classification of street youth since being accepted by UNICEF in 1986 [3], very little work has been done to characterize these sub-populations with consequent debates on how to name and describe street-connected youth [4], [5]. It has been suggested that street involvement may be a gradient that begins with a child first going to the street to support his or her family, eventually engaging in less and less family contact until he or she is living and working on the street full-time [6]. However, knowledge on whether and to what extent these transitions exist, the effect of experiences causing homelessness on the degree of street-connectedness, among other important questions related to this population, is limited due to a dearth of longitudinal studies on this population [7]. The financial independence that some youth are able to attain through street life has been identified as a factor contributing to a high attrition rate from rehabilitation programs [7], but few studies have attempted to characterize how street youth obtain and allocate money. Further, while factors motivating youth to adopt street life, such as poverty, domestic conflict, civil conflict, substance use in the home, and, to a lesser extent, orphanhood have been investigated, the vast majority of these studies have been done primarily on male populations with little to no attention given to females or with any gender analysis or analysis of how these reasons influence the level of involvement on the street [8]–[11].

The main objective of this analysis was therefore to describe the social and economic characteristics of street youth in Eldoret, Kenya, and to examine variations in these factors by gender and level of street involvement. These data may assist program implementers and policy-makers in developing tailored interventions for this vulnerable population.

## Methods

### Human Subjects Protection

This study received ethical approval from the Kenyan Institutional Research and Ethics Committee of Moi Teaching and Referral Hospital and Moi University College of Health Sciences (Eldoret, Kenya), the Indiana University Institutional Review Board (Indianapolis, USA), and the Miriam Hospital Institutional Review Board (Providence, USA). Approval for the study was also obtained from the District Children's Officer (DCO) in Eldoret. Recruited individuals interested in participating in the study were either accompanied or referred to the study clinic by the outreach worker for screening and consenting. If an individual was noted to be impaired as a result of substance use at the time of contact with the outreach worker, they were asked to delay going to the clinic until they were no longer ‘high’. At the study clinic, all prospective participants underwent a comprehension assessment to ensure the youth understood the nature of their participation in a research study, the study procedures, and their right to withdraw at any time or withhold any answers [12]. Prospective participants failing this comprehension assessment because of substance use were invited to return at another time and informed of the reason they were not enrolled in the study. Those prospective participants passing this screening phase were enrolled. Participants over 18 provided written informed consent. Those under 18 provided written assent in the presence of a child advocate, with the District Youth's Officer providing general written consent for their participation as de facto guardian. There were two prospective participants who failed to meet the criteria for understanding and were not enrolled. None refused participation in the study.

### Study Design

This is a secondary analysis of data collected for a cross-sectional study on the sexual health of street-connected youth [13].

### Study Setting

Eldoret is a rapidly growing city in western Kenya with an estimated population of 289,380, making it Kenya's fifth largest city [14]. Located within Eldoret are Moi University School of Medicine (Kenya's second largest medical school), Moi Teaching and Referral Hospital (MTRH), and the Academic Model Providing Access to Healthcare (AMPATH) Program, a large HIV care and treatment program [15], [16]. Eldoret is the administrative center of Uasin Gishu County, where 51.8% of the population lives under Kenya's poverty line, exceeding the national average of 47.2% [14].

### Study Population

To participate in this study, street youth had to meet the following inclusion criteria: aged 12–21, living in Eldoret, Kenya, spending days only or days and nights on the street, and able and willing to consent or assent. Participants spending only their days on the street were designated part-time street youth while those on the streets day and night were designated full-time street youth.

### Recruitment and Enrolment

Recruitment and enrollment was conducted from September 2011–June 2012. Youth were informed about the study through community networks, street youth support services, contacts of researchers, and by street outreach workers familiar with the population. Participants were recruited and enrolled by the street outreach workers using convenience sampling until the target sample size of 200 participants was met. Prospective participants were recruited from known ‘hang-out spots’ and places where children sleep (known locally as ‘barracks’) during the day. To address gender disparity, participants were also recruited and enrolled at Berur, an Eldoret community organization that works specifically with street girls. The study was conducted at a study clinic for high-risk youth located at Moi Teaching and Referral Hospital Berur, all of which provide healthcare or services to street youth.

### Measures and Sources of Data

Study personnel interviewed each participant privately and administered a structured questionnaire. Data were manually collected. Interviews were approximately 45 minutes in length and conducted in Kiswahili or English. The clinical encounter captured data on sociodemographics: gender (male/female), age category (12–15, 16–18, 19 years and above), parental vital status (both alive, mother died, father died, both died), person(s) they were living with prior to coming to the street, educational history (ever gone to school (yes/no), desire to return to school (yes/no). Characteristics related to their street-involvement were also gathered, including whether they were on the streets part-time or full-time, cumulative time spent on the street (1–2 years, 2–5 years, >5 years), arrest history (yes/no), jail history (yes/no), and whether they have been to a rehabilitation center (yes/no). The variables pertaining to social support were what their major source of social support was (family member, street friend, non-street friend, pastor/teacher/doctor, non-governmental or community-based organization (NGO)), and belonging to a street gang or social group (yes/no). The environmental assessment section of the questionnaire collected information on amount of money earned per day (<50 KES, 50–100 KES, >100 KES), things purchased with available money (food, drugs or alcohol, send money home, other), and first priority for available money (same). For sources of income and things purchased, participants were allowed to select as many responses as applicable. Primary dependent variables of interest were: 1) history of arrest and detention (ever, yes/no); 2) first priority for spending money (food vs. all other commodities); 3) major source of support (family members, friends on the street, other); 4) primary reason for coming to the street (poverty at home, alcoholism or violence in the home, other).

### Data Analysis

Data from the questionnaire were hand-entered into EpiInfo software (Version 6) following initial checks for errors and inconsistencies. Data cleaning was conducted looking for outliers, inconsistencies, and missing or wrong data. Categorical variables were summarized using proportions while continuous variables were examined using means and medians together with standard deviation and inter-quartile range (IQR), respectively. The 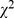
test was used to test for associations between categorical and dichotomous variables; Fisher's exact test was used alternatively if some cells had expected value of less than 5.

Primary dependent variables were selected based on their priority in terms of potential program or policy relevance, their statistical significance in bivariate analyses, and feasibility in a logistic regression (e.g. heterogeneity in response). Non-dichotomous variables of interest were dichotomized based on relevant cut-offs (for continuous variables) or categories (for categorical ones). Multivariable analysis used both binary (for binary variables) and multinomial (for nominal variables) logistic regression models to examine the association of gender and level of street involvement with these primary dependent variables of interest, adjusting for age and length of time on the street. All analyses were conducted using SAS version 9.3.

## Results

Of the 200 street youth enrolled in the study, 41% were female with a mean age of 16.3 years. Overall, 71% of the study population was on the street full-time although this varied significantly by gender: 85% of boys were on the street full-time compared to 52% of girls (p<0.001).

### Sociodemographic characteristics

Table 1 summarizes the sociodemographics of the study population, disaggregated by gender and level of street-involvement. Males tended to be younger than females (51% of boys were aged 12–15 years compared to 32% of girls); there was no significant difference in age between those on the street full-time and part-time. A majority of both genders had a history of some school attendance (87% boys, 79% girls), with more boys than girls saying that they would like to return to school given the chance (73% boys, 58% girls). More girls than boys said that both of their parents were deceased (19% boys, 35% girls), and more youth on the street full-time reported being double orphans (28%) compared to those on the street part-time (18%). The largest proportion of both sexes said they lived with their mother only before coming to the street (30% boys, 36% girls). The largest proportion of part-time street youth reported living with their mother only prior to street involvement (44%); however full-time street youth most commonly reported having lived with family other than their parents (35%).

**Table 1:** Sociodemographic characteristics of the study population by gender and level of street involvement (N = 200).

	Gender						Level of street involvement						
	Male n = 119; n (%)	Female n = 81; n (%)			P-value		Part-time n = 57; n (%)		Full-time n = 143; n (%)			P-value	
**Sex**													
Male	-----	-----			Not applicable		18 (31.6)		101 (70.6)			**<0.001**	
Female	-----	-----					39 (68.4)		42 (29.4)				
Missing	0 (0%)	0 (0%)					0 (0.0)		0 (0.0)				
**Age group (years)**													
12–15	61 (51.3)	26 (32.1)			**0.007**		29 (50.9)		58 (40.6)			0.184	
>15	58 (48.7)	55 (67.9)					28 (49.1)		85 (59.4)				
Missing	0 (0.0)	0 (0.0)					0 (0.0)		0 (0.0)				
**Has attended school**													
Yes	104 (87.4)	64 (79.0)			0.112		48 (84.2)		120 (83.9)			0.959	
No	15 (12.6)	17 (21.0)					9 (15.8)		23 (16.1)				
Missing	0 (0.0)	0 (0.0)					0 (0.0)		0 (0.0)				
**Wants to return to school**													
Yes	87 (73.1)	47 (58.0)			**0.019**		41 (71.9)		93 (65.0)			0.201	
No	31 (26.1)	29 (35.8)					13 (22.8)		47 (32.9)				
Don't know	1 (0.8)	5 (6.2)					3 (5.3)		3 (2.1)				
Missing	0 (0.0)	0 (0.0)					0 (0.0)		0 (0.0)				
**Parent vital status**													
Single orphan	64 (53.8)	33 (40.7)			0.032		29 (50.9)		68 (47.6)			0.267	
Double orphan	22 (18.5)	28 (34.6)					10 (17.5)		40 (28.0)				
Both alive	33 (27.7)	20 (24.7)					18 (31.6)		35 (24.5)				
Missing	0 (0.0)	0 (0.0)					0 (0.0)		0 (0.0)				
**Persons lived with before coming to the street**													
Mother only	35 (29.7)	29 (35.8)			0.367		25 (44.6)		39 (27.3)			0.093	
Father only	9 (7.6)	3 (3.7)					1 (1.8)		11 (7.7)				
Both parents	29 (24.6)	17 (21.0)					14 (25.0)		32 (22.4)				
Other relatives	30 (25.4)	25 (30.9)					12 (21.4)		43 (30.1)				
Well-wishers	2 (1.7)	3 (3.7)					1 (1.8)		4 (2.8)				
Father/stepmother, mother/stepfather	13 (10.9)	4 (4.9)					3 (5.3)		14 (9.8)				
Missing	1 (0.8)	0 (0.0)					0 (0.0)		0 (0.0)				

### Characteristics of street-involvement

Table 2 details characteristics of street-involvement among the study population. Poverty was the most cited reason for leaving home by females (58%) and was the second most cited reason by males (30%). Alcoholism or violence was the most common reason for boys leaving home, and the second most common reason for girls (37% boys, 15% girls). A large fraction of both part-time and full-time street youth left home citing poverty (61% part-time, 34% full-time), but an equally large proportion of full-time street youth reported leaving home as a result of alcoholism or violence (36%, compared to 7% for part-time street youth). Few youth reported leaving home as a result of civil conflict (0% part-time, 4% full-time). The majority of both sexes reported being on the street full-time (85% of boys, 52% of girls). A majority of both genders had been on the street for more than 2 years, with a larger fraction of males compared to females in this category (82% boys v. 70% girls). In terms of street involvement, the full-time street youth had been on the streets longer, with 82% saying they had been on the streets for more than 2 years, compared with 65% of part-time street youth.

**Table 2:** Street-involvement characteristics of the study population by gender and level of street involvement (N = 200).

	Gender			Level of street involvement		
	Male n = 119; n (%)	Female n = 81; n (%)	P-value	Part-time n = 57; n (%)	Full-time n = 143; n (%)	P-value
**Major reason for coming to the street**						
Poverty in the home	36 (30.2)	47 (58.0)	**<0.001**	35 (61.4)	48 (33.6)	**<0.001**
Alcoholism, violence, or abuse in the home	47 (39.5)	12 (14.8)		4 (7.0)	55 (38.5)	
Boredom/abandoned	22 (18.5)	10 (12.4)		6 (10.5)	26 (18.2)	
Peer pressure/orphaned/thrown out because of stealing	14 (11.8)	12 (14.8)		12 (21.1)	14 (9.8)	
Missing	0 (0.0)	0 (0.0)		0 (0.0)	0 (0.0)	
**Daily time spent on the street**						
Part-time	18 (15.1)	39 (48.2	**<0.001**	—	—	Not applicable
Full-time	101 (84.9)	42 (51.8)		—	—	
Missing	0 (0.0)	0 (0.0)		—	—	
**Cumulative time on the street**						
1-2 yrs	22 (18.5)	24 (29.6)	0.177	20 (35.1)	26 (18.2)	**0.036**
2-5 yrs	50 (42.0)	28 (34.6)		18 (31.6)	60 (42.0)	
>5 yrs	47 (39.5)	29 (35.8)		19 (33.3)	57 (39.9)	
Missing	0 (0.0)	0 (0.0)		0 (0.0)	0 (0.0)	
**Major source of support**						
Family member	15 (12.6)	23 (28.4)	**0.004**	24 (42.1)	14 (9.9)	**<0.001**
Street friend	33 (28.2)	10 (12.4)		3 (5.3)	40 (28.4)	
Non-street friend	11 (9.4)	4 (4.9)		3 (5.3)	12 (8.5)	
Pastor/teacher/doctor	6 (5.0)	1 (1.2)		4 (7.0)	3 (2.1)	
NGO	52 (44.4)	43 (53.1)		23 (40.4)	72 (50.4)	
Missing responses	2 (1.7)	0 (0.0)		0. (0.0)	2 (1.4)	
**Belongs to a gang or street social group**						
Yes	117 (98.3)	59 (72.8)	**<0.001**	38 (66.7)	138 (96.5)	**<0.001**
No	2 (1.7)	22 (27.2)		19 (33.3)	5 (3.5)	
Missing	0 (0.0)	0 (0.0)		0 (0.0)	0 (0.0)	
**Ever been arrested**						
Yes	90 (75.6)	45 (56.6)	**0.003**	27 (47.4)	108 (75.5)	**<0.001**
No	29 (24.4)	36 (44.4)		30 (52.6)	35 (24.5)	
Missing	0 (0.0)	0 (0.0)		0 (0.0)	0 (0.0)	
**Been to jail or juvenile detention**						
Yes	81 (68.1)	37 (45.7)	**0.002**	25 (43.9)	93 (65.0)	**0.006**
No	38 (31.9)	44 (54.3)		32 (56.1)	50 (35.0)	
Missing	0 (0.0)	0 (0.0)		0 (0.0)	0 (0.0)	
**Been to rehabilitation center or children's home**						
Yes	75 (63.0)	53 (65.4)	0.728	32 (56.1)	96 (67.1)	0.144
No	44 (37.0)	28 (34.6)		25 (43.9)	47 (32.9)	
Missing	0 (0.0)	0 (0.0)		0 (0.0)	0 (0.0)	

More girls than boys and more part-time than full-time street youth reported to be primarily supported by a family member (28% girls, 13% boys; 42% part-time, 10% full-time). Boys and full-time street youth said they were primarily supported by street friends (12% girls, 28% boys; 5% part-time, 28% full-time). Approximately half of the entire population, both girls and boys and full-time and part-time street youth, reported that non-governmental organizations were their major source of support, while less than 10% of any category reported that a pastor, teacher or doctor supported them.

Membership in a street social group or gang was more common amongst boys (98%) than girls (73%), and amongst. full-time (97%) than part-time street (67%) youth. By gender, 76% and 68% of boys reported having been arrested or jailed, respectively, compared to 57% and 46% of girls. By street involvement, 76% and 65% of full-time street youth said that they had been arrested or jailed, respectively. The corresponding numbers for part-time street youth were 47% and 44%.

### Economic Characteristics

Table 3 summarizes the reported sources of money for the study population, and spending habits and priorities. Large fractions of both males and females reported begging (53% and 64%, respectively) and casual labor (40% and 53%) as sources of money. Other significant income generating activities for males were recycling metals (73%), carrying luggage (64%), and watching cars (45%). Overall, males had more diverse sources of income when compared with the female group. Significantly more females than males reported involvement in prostitution (15% females, 1% males). In addition to begging, both part and full-time street youth reported high involvement in a wide variety of income generating activities, but full-time street youth had a larger fraction engaged in informal employment, including recycling various materials, watching cars, and carrying luggage (15–60% full-time, 7–25% part-time).

**Table 3:** Economic characteristics of the study population by gender and level of street involvement (N = 200).

	Gender		Level of street involvement	
	Male	Female	Part-time	Full-time
	n = 119; n (%)	n = 81; n (%)	n = 57; n (%)	n = 143; n (%)
**Sources of money**				
Begging	63 (52.9)	52 (64.2)	31 (54.4)	84 (58.7)
Stealing or pickpocketing	2 (1.7)	1 (1.2)	3 (5.3)	0 (0.0)
Metals recycling	87 (73.1)	9 (12.4)	10 (17.5)	86 (60.1)
Plastics recycling	32 (26.9)	10 (12.4)	6 (10.5)	36 (25.2)
Papers recycling	23 (19.3)	3 (3.7)	4 (7.0)	22 (15.4)
Selling plastic bags	2 (1.7)	2 (2.5)	0 (0.0)	4 (2.8)
Selling drugs	1 (0.8)	2 (2.5)	1 (1.7)	2 (1.4)
Watching cars	53 (44.5)	3 (3.7)	11 (19.3)	45 (31.5)
Carrying luggage and bags	76 (63.9)	6 (7.4)	14 (24.6)	68 (47.6)
Casual labor	47 (39.5)	43 (53.1)	24 (42.1)	66 (46.2)
Prostitution	1 (0.8)	12 (14.8)	5 (8.8)′	8 (5.6)
Given money by relatives/friends	2 (1.7)	10 (12.4)	7 (12.3)	5 (3.5)
Making ornaments	0 (0.0)	7 (8.6)	7 (12.3)	0 (0.0)
Collecting charcoal/cigarettes	2 (1.7)	2 (2.5)	0 (0.0)	4 (2.8)
Other	9 (7.6)	7 (8.6)	6 (10.5)	10 (17.5)
Missing	0 (0.0)	0 (0.0)	0 (0.0)	0 (0.0)
**Amount earned daily (KES)**				
<50	12 (10.1)	22 (27.2)	18 (31.6)	16 (11.2)
50-100	51 (42.9)	21 (25.9)	17 (29.8)	55 (38.5)
100-500	55 (46.2)	33 (40.7)	18 (31.6)	70 (49.0)
>500	1 (0.8)	0 (0.0)	0 (0.0)	1 (0.7)
Don’t know	0 (0.0)	5 (6.2)	4 (7.0)	1 (0.7)
Missing	0 (0.0)	0 (0.0)	0 (0.0)	0 (0.0)
**Things bought with earned money**				
Food	118 (99.2)	68 (84.0)	44 (77.2)	142 (99.3)
Glue, alcohol, or other drugs	75 (63.0)	23 (28.4)	13 (22.8)	85 (59.4)
Take money home	24 (20.2)	21 (25.9)	29 (50.9)	16 (11.2)
Clothes/shoes/soap/lotion	46 (38.7)	35 (43.2)	14 (24.6)	67 (46.9)
Save for other necessities	10 (8.4)	6 (7.4)	4 (7.0)	12 (8.4)
Rent/bus fare	6 (5.0)	1 (1.2)	1 (1.8)	6 (4.2)
Other	18 (15.1)	6 (7.4)	7 (12.3)	17 (11.9)
Missing	0 (0.0)	0 (0.0)	0 (0.0)	0 (0.0)
**First priority for available money**				
Food	107 (89.9)	63 (77.8)	41 (71.9)	129 (90.2)
Glue, alcohol, or other drugs	8 (6.7)	2 (2.5)	1 (1.8)	9 (6.3)
Take money home	2 (1.7)	6 (7.7)	7 (12.3)	1 (0.7)
Other	2 (1.7)	6 (7.7)	4 (7.0)	4 (2.8)
Refused to answer	0 (0.0)	1 (1.2)	1 (1.8)	0 (0.0)
Missing	0 (0.0)	3 (1.3)	3 (5.3)	0 (0.0)

Males also earned, on average, more than females, with 90% of males earning more than 50 KES per day (0.59 USD), while only 73% of females earned a similar amount. Full-time street youth earn more on average than their part-time counterparts, with 89% of them earning >50 KES daily, compared with 68% of part-time street youth.

When money was available, a majority of all groups said that they bought food (99% boys, 84% girls; 99% full-time, 77% part-time). While 63% of boys and 60% full-time street youth said they would buy drugs with their money, only 28% of girls and 23% of part-time street youth said the same. Part-time street youth were much more likely to say they took a portion of their money home (51% part-time, 11% full-time). When asked about which choice took first priority when money was available, 90% of boys and full-time street youth picked food, compared to 78% of girls and 72% of part-time street youth. Glue, alcohol, and other drugs were not cited as a priority by a majority of any group.

### Multivariable Analyses

Tables 4 and 5 describe the association of gender and level of street-involvement with key social and economic characteristics using both binary (Table 4, for binary effects) and multinomial logistic regression for non-ordered categorical effects (Table 5). Compared with part-time street youth, full-time street youth were more likely to have been arrested (Adjusted Odds Ratio [AOR]: 2.33, 95% Confidence Interval [95% CI]: 1.01–5.35), name food as their first spending priority (AOR: 2.57, 95% CI: 1.03–6.45), to report that friends on the street were their major source of support (AOR: 3.59, 95% CI: 1.01–12.82), and to have left home due to alcoholism or violence (AOR: 5.54, 95% CI: 1.67–18.34). Compared with females, males were more likely to have ever been arrested (AOR: 2.66, 95% CI: 1.14–6.18), and to have ever been jailed (AOR: 3.22, 95% CI: 1.47–7.02).

**Table 4:** Unadjusted (UOR) and Adjusted Odds Ratios (AOR) and 95% Confidence Intervals (CI) for associations of gender and level of street involvement with key social and economic indicators among street-connected youth aged 12–21 (N = 200).

	UOR	95%CI	AOR	95%CI
**Ever arrested (n = 200)**				
Gender (male vs. female*)	1.80	0.94–3.47	2.66	1.14–6.18
Street involvement (full vs. part-time*)	2.78	1.40–5.51	2.33	1.01–5.35
**Ever been to jail (n = 200)**				
Gender (male vs. female*)	2.11	1.13–3.91	3.22	1.47–7.02
Street involvement (full vs. part-time*)	1.82	0.93–3.55	1.38	0.63–3.02
**First priority for money (food vs. all other responses) (n = 197)**				
Gender (male vs. female*)	1.58	0.65–3.81	1.28	0.50–3.27
Street involvement (full vs. part-time*)	2.48	1.02–6.04	2.57	1.03–6.45

**(**Binary multivariable logistic regression analysis for the association of gender and level of street involvement with binary indicators adjusted for age and length of time on the street).

*reference category.

**Table 5:** Unadjusted (UOR) and Adjusted Odds Ratios (AOR) and 95% Confidence Intervals (CI) for associations of gender and level of street involvement with key social and economic indicators among street-connected youth aged 12–21 (N = 200).

Covariate	Dependent variable	UOR (95%CI)	AOR (95%CI)
	**Major source of support (n = 198)**		
Gender (male vs. female*)	family member	0.74 (0.32–1.68)	0.66 (0.27–1.63
Gender (male vs. female*)	friend on the street	1.79 (0.79–4.08)	2.15 (0.89–5.21)
Street status (full vs. part-time*)	family member	0.22 (0.10–0.51)	0.24 (0.10–0.55)
Street status (full vs. part-time*)	friend on the street	3.79 (1.06–13.55)	3.59 (1.01–12.82)
	**Major reason for coming to the street (n = 200)**		
Gender (male vs. female*)	poverty	0.51 (0.25–1.04)	0.52 (0.24–1.12)
Gender (male vs. female*)	violence	1.71 (0.72–4.08)	1.78 (0.70–4.56)
Street status (full vs. part-time*)	poverty	0.77 (0.36–1.63)	0.78 (0.36–1.68)
Street status (full vs. part-time*)	violence	5.24 (1.60–17.17)	5.54 (1.67–18.34)

(Multinomial logistic regression analysis for the association of gender and level of street-involvement with non-ordered categorical indicators adjusted for age and length of time on the street.).

*Reference category.

## Discussion

These findings suggest a heterogeneous yet highly vulnerable street youth population in this resource-constrained setting. The social and economic characteristics of each subgroup are distinct, but all of the youth profiled here appear resourceful, employing a wide range of strategies to meet their needs on the street. Such knowledge can assist in developing interventions targeted towards the diverse strengths and vulnerabilities of this population.

### Gender

It has previously been hypothesized that, for Kenyan girls, adopting street life indicates disintegration of the family unit and societal victimization [8]. While we did not observe that girls left home primarily as a result of alcoholism or violence, the girls had fewer resources and coping skills when compared with their male counterparts. The girls in our study were more likely to be double orphans and were less likely to have schooling to fall back on or to join street social networks. Our female participants reported less daily income compared with boys, consistent with past research that found street girls had fewer economic opportunities and earned less money than boys for performing similar activities [17]. The frequency of girls reporting engagement in prostitution is worrisome and potentially underreported, indicating a unique vulnerability of this subpopulation, especially in the context of otherwise limited options for income generation.

Male street youth by no means have an easy lifestyle, experiencing significantly increased interaction with legal authorities and the detention system. However, the responses of the boys in our study supports the ideas set forth by previous research, portraying a group of boys who, in the face of hardship, are able to develop diverse strategies for earning money and form strong support networks of friends [8].

It should be noted that the gender-specific trends observed here are likely not entirely unique to the street youth population. For example, the general prison population in Eldoret is predominantly male; females in the general population have fewer opportunities for diversified income generation compared with males [18], [19]. Regardless, these gender differences are important to understanding the needs and circumstances of this particular population, and should be considered when devising reintegration and rehabilitation strategies for street youth.

### Level of street involvement

The data suggest that full-time street youth have used the streets to replace a home life and work to take care of themselves. These youth, isolated from family, generally join social groups for support (86% of boys and 82% of girls). A previous study of the Eldoret street child population found that full-time street youth maintain a large group of friends which provide functions expected from a family [20]. We see full-time street youth working more, earning more, and preferentially purchasing vital items such as food more commonly than the part-time street youth. Despite the fact that a large portion of full-time street youth do purchase drugs, it is important to note how few of them reported a preference for purchasing drugs—survival, rather than substance use, appears to be paramount to these youth, a theme consistent in the literature [21]–[23].

Because of their focus on survival and strong dependence on peer networks, full-time street youth are the distinctly more vulnerable population. This is particularly in evidence given the high likelihood that they came to the streets because of the need to leave a home environment of violence and/or alcoholism, and the high probability of being arrested and jailed. Compared with part-time street youth, a larger proportion of full-time street youth purchase drugs, presumably for their own use. Substance use within this population has been identified both as a coping mechanism as well as a component of a shared street culture that exists among street social groups [9], [24].

### Policy Implications

There are several policy implications to these findings, particularly relevant for local and national governments. First, we found that very high proportions of the study population, both male and female, have been arrested and spent time in jail or detention facilities. The incarceration of youth in general and females in particular is known to be fraught with poor conditions, breaches of their human rights, and a distinct lack of emphasis on rehabilitation [25], [26]. Researchers, national bodies, and international human rights groups have reported that police and security forces have abused children on the streets of cities all over the world [27]. Additionally, detention of youth is known to be associated with poor psychological outcomes, impaired reintegration into society, and a high rate of recidivism [28]. For youth who already come from an environment of violence and neglect, the consequences of detention on top of this may create serious obstacles to societal reintegration that are insurmountable. Without a comparison group from the general population, the increase in interaction with legal authorities experienced by street youth is difficult to quantify. However, regardless of relative frequency, the high proportion of youth arrested and incarcerated seen in this population is a major policy issue that needs to be urgently addressed. Local and national governments must find alternative strategies to handling the ‘epidemic of street youth’ in a way that maximizes protection and rehabilitation, and minimizes detention and abuse.

The global population of street-connected youth continues to increase with few improvements in their situation [2], [29]–[32]. Our results challenge the commonly held assumption that many youth end up on the street as a result of ethnic violence, delinquency or boredom [6], [33], [34]. The fact that the majority of youth in our study cite poverty, violence and alcoholism as the primary reasons for leaving home, points to the need for policies that help to support families to care for the children they are responsible for and through more extensive child-protection initiatives. Preventing youth from having to come to the street should be the top priority for policy makers.

Presently, local governments in Kenya are endeavoring to forcibly return street youth back to where they come from, a process that can be thought of as ‘universal reunification’. Universal reunification of street youth with their families (or whoever is found when they are taken there) without a risk assessment of the home environment has shown limited benefit and can be detrimental [35]–[37]. Whether youth leave home as a result of abusive conditions or as a result of poverty, our data suggest that the vast majority end up on the street because of seriously adverse home environments. Local governments must consider the capacity of households and communities for reintegrating these youth without additional support. A simplistic repatriation strategy is likely to fail. Moreover, the current trend by local governments to deter or prevent non-governmental (NGO) and community-based organizations from supporting street-connecting youth is likely to have a detrimental impact on youth given that half of the population in our study reported that their primary source of support was from these agencies. Local and national governments should be encouraged to work with academic and NGO stakeholders to find the best preventive and rehabilitation/reintegration strategies that are in the best interests of the youth.

### Strengths & Limitations

One of the major strengths of this study is its relatively large proportion of female street youth, one of the largest in current literature. Another is the high rate of response to all portions of the interview questionnaire by the participants. Both may be attributed to the strong trust and established relationship between our study team and the street-connected youth community in Eldoret [15], [38]–[41], including our use of street-based outreach workers and their assistance of the youth in matters unrelated to research. Our study also goes beyond traditional analyses of youth's motivations for adopting street life and their subsequent risk of abusing substances [42]. This population is understudied and there are few data to guide program implementers and policy makers; this study helps to fill that gap. This analysis does not address the sexual behaviors, substance use or mental health of this population. These are extremely important issues that we are addressing through separate analyses, studies, and publications [13], [43]–[47].

One of the main limitations of this study is inherent to the use of a convenience sample and cross-sectional data. For example, our study has identified a much larger proportion of street-connected youth being on the street full-time compared to what has traditionally been reported [3], [5]. As our data were based on a cross-sectional convenience sample, caution should be used in generalizing from this to the whole population of street youth. This study has the potential for selection bias toward those youth easiest to access and a failure to enroll those youth more difficult to reach. Additionally, due to the dynamic and transient nature of the street youth population, their characteristics and experiences are constantly in flux; a convenience sample of cross-sectional data from these individuals may not reflect the full story or may become quickly outdated. There may have been some responder bias if participants answered questions according to what they thought the interviewer wanted to hear. However, it should be noted that the interviewer, a medical officer, was well-trained in interview techniques and participants had nothing to gain or lose from the nature of their responses. The cross-sectional design has inherent limitations, including an inability to draw conclusions about the temporal nature of the exposures and outcomes examined. The cultural and geographical heterogeneity within resource-constrained settings means that it cannot be assumed that the trends observed in the Eldoret population are immediately generalizable to all street youth. The categorization of whether or not a child spends nights on the street utilizes a simple binary metric (full-time, part-time). While widely accepted, distilling the varied experiences of street children into these two categories has been criticized as an oversimplification that fails to fully address family ties and entrenchment in street life [48]. While we accept this limitation, these data nevertheless provide a more nuanced perspective into the social and economic characteristics of street-connected youth in this setting compared to most other available data on them. Finally, by enrolling youth ages 12 and above, we excluded young children, of whom there are many on the street. Caution should be used in interpreting these data.

## Conclusions

These data suggest that this street youth population is heterogeneous and resourceful, yet highly vulnerable. Despite frequently reported statistics connecting Kenya's street youth population with ethnic violence or boredom, few youth actually reported these as reasons for adopting street life. The majority of youth who participated in this study left home either because of poverty or alcoholism and violence in the home. In either case, a failure of familial support, be it financial, emotional, or both appears to have driven them to search for something more on the streets. These data emphasize the urgent need for governments and other stakeholders seeking to intervene on behalf of these youth to address the various social and economic factors pulling them to the streets and obstructing their societal reintegration. A fundamental and detailed understanding of this population's heterogeneity will help to inform social reintegration policies and programs by recognizing and addressing the diverse needs of these young people.
